# Beyond the incubator: applying a “one health” approach in the NICU

**DOI:** 10.1038/s41390-024-03534-4

**Published:** 2024-08-30

**Authors:** Daniel O’Reilly, Alison Livada, Laurie Steiner, Richard J. Drew, Naomi Mc Callion

**Affiliations:** 1 Department of Neonatology, Rotunda Hospital, Dublin 1, Ireland; 2 School of Biomolecular and Biomedical Science, University College Dublin, Dublin 4, Ireland; 3 Medical Scientist Training Program, School of Medicine and Dentistry, University of Rochester, Rochester, NY USA; 4 Department of Pediatrics, University of Rochester School of Medicine and Dentistry, Rochester, NY USA; 5 Irish Meningitis and Sepsis Reference Laboratory, Children’s Health Ireland at Temple Street, Dublin, Ireland; 6 Clinical Innovation Unit, Rotunda Hospital, Dublin 1, Ireland; 7 Department of Clinical Microbiology, Royal College of Surgeons in Ireland, Dublin, Ireland; 8 Department of Paediatrics, Royal College of Surgeons in Ireland, Dublin, Ireland

## Abstract

**Abstract:**

A “one health” approach recognises that human health, animal health and planetary health are closely interlinked and that a transdisciplinary approach is required to fully understand and maintain global health. While, by necessity, Neonatal Intensive Care has traditionally focused on the acutely unwell newborn, the avoidance of long-term harm is core to many management decisions. The COVID 19 pandemic and climate crisis have brought into sharp relief the importance of a “one health” approach as part of long-term health promotion in the holistic care of neonates, who may survive to experience the burden of future environmental crises. This narrative review seeks to integrate what we know about “one health” issues in the neonatal intensive care unit, notably antimicrobial resistance and climate change, and suggest “everyday changes” which can be utilised by practitioners to minimise the impact of neonatal intensive care on these global health issues. Many of the changes suggested not only represent important improvements for planetary health but are also core to good neonatal practice.

**Impact:**

- Neonatal patients are likely to bear the burden of future environmental crises including pandemics and climate related disasters.
- While the focus of intensive care practitioners is acute illness, awareness of “one health” problems are important for our smallest patients as part of preventing long-term harm.
- High quality neonatal care can benefit both the planet and our patients.

## Introduction

The “One Health” approach recognises that the health of people, animals and the environment are closely interlinked, and that maintaining global health requires a transdisciplinary approach.^1,2^ Initially conceived during the SARS CoV-1 outbreak in 2002-2004, “One Health” represents a durable approach to many of the global issues that we now face, including antimicrobial resistance and climate change. Its importance was further underlined by the emergence of SARS CoV-2/COVID 19 in 2019/2020 and the resulting global pandemic.

Whilst the neonatal intensive care unit (NICU) has traditionally focused on the “here and now” of the acutely unwell neonate, minimising life-long morbidities and challenges for survivors is also key to good neonatal practice. A “One Health” approach in the NICU is increasingly important as part of this life-long approach. Today’s neonatal patients, expected to live for over 80 years and will bear the brunt of global issues such as the climate crisis and antimicrobial resistance, as well as the emergence of novel zoonoses.^3^

In this narrative review, we will examine the ways in which NICU contributes to, and is affected by, “One Health” issues. Neonatologists are frequent prescribers of antibiotics, with approximately 80% of patients admitted to NICU receiving at least one dose of antibiotic.^4^ Multi-drug resistant organisms (MDROs) are on the rise globally, and a substantial proportion of neonates are colonised with an MDRO.^5^ We will discuss current practices which aim to reduce the use of antibiotics in the NICU, and future steps required to reduce overall antibiotic exposure in the neonatal period. A brief discussion of the implications of animal health on common NICU pathogens will underline the interconnected nature of our planet and the importance of thinking beyond the doors of the hospital.

Climate change will have a tremendous impact on the lives of our smallest patients, and yet their NICU stay may exacerbate the problem.^6^ Infant feeding practices, waste management and the structure of developmental clinics all play a potentially important role as we attempt to achieve carbon neutrality in the near future. Climate change will have profound impacts on how neonatal care is delivered in both low- and middle- income countries (LMICs) and higher income countries (HICs) due to the direct effects of changing temperatures and climate patterns, and due to the spread of vector borne diseases to areas which were previously unaffected, or only seasonally affected.

## Antibiotic use and resistance in the NICU

Antibiotics are the most commonly used medication in NICU. Neonatal sepsis presents with subtle, non-specific symptoms initially and may insidiously progress to a severe inflammatory syndrome with resulting neurodevelopmental deficit and/or death. As such, neonatal practitioners often feel compelled to treat symptomatic neonates, or those with a high risk of sepsis, with empiric antibiotics whilst awaiting culture results.^5^ Moreover, despite the sensitivity of many common neonatal bacterial pathogens to narrow spectrum antibiotics, escalation to broader spectrum agents such as third generation cephalosporins and extended spectrum penicillins/beta-lactams is common.^7^ This practice is unsatisfactory for a number of reasons. First, exposure to prolonged, broad-spectrum antibiotics in infants with negative cultures increases the risk of developing necrotising enterocolitis (NEC), invasive candidiasis, late-onset sepsis and even death.^5^ The development of NEC and sepsis trigger treatment with further broad spectrum antimicrobial agents, perpetuating the cycle. Second, early exposure to antibiotics has been associated with the development of atopy and allergy.^8^ The retrospective data from Zven *et al*. may have been influenced by confounding factors, however pre-clinical animal models have suggested that changes to the developing microbiota can heighten T-helper 2/T-helper 17 pathways and airway responses.^9^ This context is important from a “one health” perspective, as metered dose inhalers for asthma are one of the largest contributors to the healthcare-related carbon footprint.^10^ Thirdly, all antibiotic use contributes to the development of resistant organisms, and MDROs have a profound impact on neonatal care with increased risk of mortality, particularly in LMICs where access to targeted antibiotic therapies may not be available.^5^

Everyday changes to NICU practice could alleviate this problem. Antimicrobial stewardship reduces both the use of broad spectrum antibiotics and the number of days of therapy in neonates with negative blood cultures.^11^ Numerous approaches have been utilised to try and reduce antibiotic exposure in late preterm and term newborns, where the the individual risk of early-onset sepsis is lowest and at the gestational age where the majority of babies are born.^12,13^ Despite this there is a wide range of antibiotic utilisation in this group even in high income countries with one recent cross-sectional study describing between 1.18% and 12.45% of neonates in this group treated for early-onset sepsis.^14^ The implementation of neonatal Early-onset Sepsis calculators in some centres has significantly reduced the use of empiric antibiotics without an increase in the incidence of missed early-onset sepsis.^15^ However the lowest rate of antibiotic exposure utilising this tool is 2.6–3% with centres using serial clinical examination examination in high risk neonates were able to achieve even lower antibiotic exposure.^14^ Serial physical examination for risk assessment has been demonstrated to reduce antibiotic exposure without increasing infection mortality or affecting culture positive early-onset sepsis rates.^16^ It is worth acknowledging that this requires a change in clinical workflow and staffing commitments to perform which may be difficult to achieve outside of tertiary units in HIC. A preference for using narrow spectrum antibiotic regimens over broader spectrum approaches removes selection pressure favouring MDROs, and has been shown to be beneficial in a multicentre crossover study comparing two set regimens.^17^

The use of early-onset sepsis risk stratification strategies in LMICs is complicated by numerous factors. A greater proportion of births occur at home/in the local community, without access to modern medical infrastructure or skilled attendants.^18^ In neonates who do receive a diagnosis of culture proven early-onset sepsis, there is a greater predominance of non GBS organisms such as Staphylococcus aureus, Actineobacter spp. and Klebsiella spp.^18–20^ There is an urgent need for a practical early-onset sepsis risk stratification tool that can be used in these lower resource settings, or validation of existing risk stratification guidelines in a LMIC context.

There also remain areas for improvement in NICUs based in HICs. A recent study demonstrated that oral antibiotics for suspected culture negative sepsis were non-inferior to intravenous antibiotics in a group of “culture negative” neonates.^21^ Although this was commendable in reducing the number of intravenous access attempts on involved neonates, and allowing newborns to be discharged home in a timely manner, a more useful trial might be to discharge the same group of low-risk infants home without systemic antibiotic therapy, as outlined in the American Academy of Paediatrics (AAP) guidelines.^13^ Preterm infants often receive antibiotics due to concerns regarding chorioamnionitis, however robust evidence for the practice in this population is lacking. The NICU antibiotics and outcomes trial (NANO, NCT03997266) will hopefully provide some data to guide antibiotic prophylaxis in Extremely Low Birth Weight (ELBW) neonates, however given the very restrictive inclusion criteria and wide exclusion criteria this may directly benefit only a small population of preterm infants.^22^

Reducing the rates of “culture negative” sepsis is also an important consideration. Whilst it is often suggested that blood cultures represent a poorly sensitive tool for the detection of newborn sepsis, if an adequate inoculum is provided they represent a robust means of detection of bacteremia, where present. An inoculum of at least 0.5 ml per blood culture bottle is traditionally deemed sufficient; however using 0.5 ml instead of 1 mL inocula reduces blood culture sensitivity by 10–40%.^5^ Collecting 1 ml of blood volume can be challenging in late-onset sepsis investigations in ELBW infants with poor intravenous access, for most other neonates this higher volume should be prioritised.^23^ Moving away from the use of currently available inflammatory markers (CRP, Procalcitonin and IL-6) in decisions around commencing or escalating antibiotic therapy is warranted, given their lack of sensitivity.^13^ Studies which investigate new markers which are better “rule in” tests for neonatal sepsis should be aimed at the ELBW population, and at neonates in LMICs and lower resource settings in particular, where access to fully-equipped microbiology laboratories may be limited.

Everyday changes in practice can reduce antibiotic use, and quality improvement bundles have demonstrated that increased stewardship, use of an Early-onset Sepsis Calculator and emphasis on correct inoculant volume for blood cultures can all reduce local antimicrobial use.

## NICU and animal health

Another important component of the “One Health” model is that of animal health. Although zoonoses are rarely considered in the neonatal unit, outside of rare vertical transmission concerns, many animal reservoirs exist for common infections found in neonatal units, and these are important contributors to microbiota found in each individual unit.

Candidemia is a relatively common cause of late-onset sepsis, and is associated with high mortality in the neonatal intensive care unit. Whilst *Candida albicans* remains the most common cause of fungemia in this context, increasing numbers of non-albicans candida have been implicated in neonatal sepsis.^24^ *Nakaseomyces glabratus (*formerly *Candida glabrata)* is the second most common cause of candidemia in adult patients and an increasing problem in neonatal units where, depending on region, it may represent almost half of candidemia cases.^24^ Unlike *C. albicans*, which is primarily a human commensal, *N. glabratus* has important environmental reservoirs. One important reservoir is the yellow-legged gull in the Mediterranean, which can facilitate the dissemination of antifungal resistant *N. glabratus*.^25^ The spreading of the antifungal resistant strain is hypothesised to arise when the gulls feed on human landfill sites before flying to another area, where humans become colonised due to environmental contamination by the birds.^25^ Similarly the recent emergence of *Candida auris*, a candida species which has been implicated in severe, antifungal resistant neonatal sepsis, has been associated with increasing global temperatures, which allows it to transition from plants/wetlands and onto bird species, from where it could successfully transition to humans.^26,27^

Livestock, and overuse of antibiotics in modern agricultural practices, have had a profound influence on a number of resistant strains of bacteria which cause neonatal sepsis.^28^ Common neonatal pathogens including *E. coli, Staphylococcus spp*. and *Enterococcus spp*. all have emerging resistant strains in the context of modern intensive livestock practices.^28^ Conversely, neonatal care also has implications for both agriculture and aquaculture. Resistant group B streptococcus species have likely been transferred from humans to cattle, where they are an important cause of mastitis.^29^ Similarly GBS strains from South Asian fish farms share human GBS derived virulence factors, suggesting spill over from the human population.^30^

## Carbon impact of the NICU

Healthcare contributes approximately 4–5% of worldwide greenhouse gas production and is considered a “heavy emitting” sector. The causes are many, including a dependence on single use items which then require replacement, medical gas and propellant use which have direct greenhouse gas effects, and high energy use.^6^ There have been no direct estimates of the carbon footprint of the neonatal unit as a standalone entity, although there are estimates for other intensive care settings, but many practices within the NICU are costly from a carbon perspective, with high energy demands and waste production a feature of modern intensive care units.^31–33^

Electricity demands in a NICU are substantial, with incubators requiring power as well as ventilators and artificial lighting. Some everyday family centred practices can help reduce these carbon costs such as regular skin-to-skin contact and increased natural lighting where infrastructure allows, with neurodevelopmentally appropriate lighting used to protect neonatal circadian rhythms.^34^ These should be considerations in any future NICU design, however institutional decarbonisation and movement to green energy sources are likely to be a more effective means of reducing this carbon burden.^35^

Waste management in the NICU is also a carbon intensive process with capacity for improvement in a number of domains.^36^ Processing of waste varies from jurisdiction to jurisdiction, but there are often multiple waste streams which can be disposed of using standard waste disposal methods or high-risk/biohazardous waste disposal methods. High risk waste as rule has a higher carbon footprint due to the existence of fewer facilities to process it, the higher temperatures required for incineration and extra steps which may be required to turn biohazardous waste into waste safe for disposal (chemical decontamination, autoclaving). As such, choice of waste streaming can have a 50-fold impact on carbon footprint.^37^ Despite this large impact, many clinical settings do not successfully triage refuse, with large volumes of waste suitable for standard waste disposal being placed into biohazardous/high risk streams. Evidence is sparse regarding NICU specifically but one waste management audit from adult ICU suggested that up to 60% of ICU-related waste could be successfully recycled.^31,36^

Clinical waste is also augmented by practices such as excessive or unnecessary testing. Clinical laboratory testing is not a carbon neutral process, with each sample being processed using sample tubes often shipped from another country prior to use and requiring the generation of biohazardous sharps for disposal.^38^ One study from Australia estimated the carbon footprint for individual laboratory tests and identified that haematological tests including full blood counts and conventional coagulation tests (Prothrombin time, Activated prothromboplastin time and fibrinogen) were the most carbon costly at 116 g/test and 82 g/test respectively. Individual biochemical tests were, on the whole, less carbon costly per item as they were often ordered in combination with other tests on the same samples.^38^ Another study from the same group estimated that the workhorses of neonatal radiological imaging, mobile chest X-ray imaging and ultrasound scanning had associated carbon costs of 0.5 g/scan.^39^

Telemedicine has become integrated into care delivery in many sectors since the COVID-19 pandemic, not least neonatal follow-up. Telemedicine has been projected to produce context dependent carbon savings of between 0.7–372 kg of CO_2_ equivalents per consultation, largely due to reduced patient travel (depending on distance)^40,41^ and has been demonstrated to reduce NICU follow-up hospital attendances in one Swedish study.^42^ Important concerns remain about patient access and optimal provision of telemedicine services, especially in providing standardised assessments through teleconferencing platforms.^43^ Despite this limitation, the use of telemedicine in the follow-up of lower risk NICU graduates, and as supplementation of the high quality care higher risk NICU graduates receive in-person, may provide a substantial reduction in carbon footprint.

Infant feeding is an important contributor to the carbon costs of neonatal and infant care. Breastfeeding, widely acknowledged as an important component of infant health particularly in neonates born preterm, has only recently been appreciated for its positive impact on planetary health. Breastfeeding neonates and infants up to 6 months exclusively has an important impact on the carbon footprint per baby, with savings of 95–153 kg/CO_2._ Important environmental gains can therefore be made by providing adequate support for mothers who wish to breastfeed both within the NICU and beyond.^44^

Many of the challenges of a greener neonatal unit are beyond the scope of an individual clinician’s practice, simple measures such as proper waste triage and supporting breastfeeding within the NICU and beyond could have a substantial impact on the carbon footprint of individual units.

## Climate change and neonatal practice

Combating climate change through everyday changes in the NICU is important for the future of our patients, changes in global temperatures will create fundamental changes in the way neonatology is practiced. Unfortunately the greatest burden of change is likely to occur in areas least equipped to combat it, in LMICs.^45^ Direct risks to young families and pregnant women include displacement secondary to meteorological disasters, which can move families away from areas of support and destroy homes and businesses. However, risks to neonatal care go beyond this. Higher temperatures have been associated with an increased risk of preterm birth, stillbirth and low birth weights in meta-analyses.^46^

Flooding and displacement have important consequences for water security, which itself has important implications for infant feeding.^45^ Whilst infants who are exclusively breastfed do not need additional water, their mothers need to remain hydrated in order to provide an adequate supply.^47^ Contaminated or dirty water sources increase the likelihood of infection in babies who are formula fed, as clean water is a prerequisite for preparing formula products.

Climate change has important implications for infections in the perinatal and neonatal period. Higher temperatures and humidity have been associated with a higher incidence of group B streptococcus carriage in one Spanish study.^48^ Common neonatal pathogens such as *Escherichia coli*, *Staphylococcus aureus* and *Klebsiella pneumonia* have demonstrated increased antibiotic resistance in areas with higher temperatures and increased population density− two issues which are likely to rise in the context of increasing temperatures and rising sea levels as less of the earth’s surface will be easily habitable by humans.^49^

Vector borne diseases are likely to have an increased geographic spread, which will have profound implications for perinatal care. Maternal malaria has a number of consequences for neonates, with high incidence of maternal anemia, premature delivery, intrauterine growth restricted (IUGR) and low birth weight infants.^50^ The relationship between rising temperatures and malaria is complex. Due to the dependence of mosquitos on standing water in order to complete their lifecycle, most modelling estimates suggest an increased population at risk with global climate changes. This effect is likely to affect both established malaria endemic areas and areas which are sub-tropic/temperate and currently malaria free.^51^ Underlining this risk is the recent report of local transmission of malaria in the southern United States in 2023.^52^ Although Malaria was eradicated in Europe as part of public health campaigns of the 20th century, rising temperatures and the continuing geographic spread of many vector species of mosquito are increasing the risk of malaria returning in those areas.^53^ This could profoundly affect maternal and neonatal management in these areas with an increase in the burden of prematurity and IUGR driving increased demands on neonatal services.

Arboviruses, or viruses spread by arthropods, are also an important perinatal infection concern. Whilst the best known example of this is Congenital Zika Virus (CZV), which is associated with potent neuroteratogenic effects, other arboviruses can have potent effects in the perinatal period.^54^ Other viruses such as Dengue and Chikungunya are associated with vertical transmission characterised by an acutely unwell neonate with thrombocytopenia and multiorgan dysfunction, mimicking bacterial/fungal septicemia.^55,56^

Climate change is likely to alter how neonatal-perinatal medicine is practiced and it is therefore incumbent on neonatal practitioners to make the small changes required to attempt to limit its affects.

## Conclusions and everyday practice changes

Human activity has had a profound impact on both planetary and animal health. Whilst in the NICU the acuity of our patient workload can sometimes limit our ability to examine global practices is, the fact is our patients will likely carry the burden of the twin threats of climate change and antimicrobial resistance after leaving our care. Table 1 highlights the practical measures individual units can take to try and improve carbon footprints generated in the NICU and reduce antimicrobial resistance in both our patients and in animals.

**Table 1:** Suggested changes to apply one health principles in the NICU.

Improving antimicrobial resistance in NICU and beyond	Improving carbon foot prints in NICU
Ensure adequate inoculums in blood cultures	Appropriate waste triaging practices with biohazardous waste being correctly identified
Stop antibiotics appropriately in culture negative infants	Appropriate support and encouragement of breastfeeding for parents who are able to do so
Reserve broader spectrum antibiotic agents for infants with proven resistant organisms	Use of telemedicine where appropriate over face to face consultations for developmental follow-up
Use of Early-Onset Sepsis risk stratification strategy to determine healthy neonates who require a septic work-up in HIC	
Continue development of novel early “positive” biomarkers for neonatal sepsis	

The “One Health” approach remains an underappreciated area of our care provision and future research should focus on how NICU care can be delivered in a sustainable way. Although some goals should be focused on improving diagnostics, such as diagnosis of sepsis, much can be achieved within current clinical practice. Fortunately, many of these everyday changes not only improve the sustainability of neonatal care but also represent best standard of care for neonatal patients. These changes allow neonatologists not only to meet our patients’ acute needs but also provide for their future beyond the incubator.
